# Parallel opsin switches in multiple cone types of the starry flounder retina: tuning visual pigment composition for a demersal life style

**DOI:** 10.1038/s41598-018-23008-y

**Published:** 2018-03-19

**Authors:** Ilaria Savelli, Iñigo Novales Flamarique, Tom Iwanicki, John S. Taylor

**Affiliations:** 1 Department of Biological Sciences, Simon Fraser University, Burnaby, British Columbia V5A 1S6 Canada; 2 Department of Biology, University of Victoria, Victoria, British Columbia V8W 2Y2 Canada; 3 Department of Biology, University of Hawai’i at Mãnoa, Honolulu, Hawai’i 96822 USA

## Abstract

Variable expression of visual pigment proteins (opsins) in cone photoreceptors of the vertebrate retina is a primary determinant of vision plasticity. Switches in opsin expression or variable co-expression of opsins within differentiated cones have been documented for a few rodents and fishes, but the extent of photoreceptor types affected and potential functional significance are largely unknown. Here, we show that both single and double cones in the retina of a flatfish, the starry flounder (*Platichthys stellatus*), undergo visual pigment changes through opsin switches or variable opsin co-expression. As the post-metamorphic juvenile (i.e., the young asymmetric flatfish with both eyes on one side of the body) grows from ~5 g to ~196 g, some single cones and one member of unequal double cones switched from a visual pigment with maximum wavelength of absorbance, λ_max_, at shorter wavelengths (437 nm and 527 nm) to one with longer λ_max_ (456 nm and 545 nm, respectively) whereas other cones had intermediate visual pigments (λ_max_ at 445 nm or 536 nm) suggesting co-expression of two opsins. The shift toward longer wavelength absorbing visual pigments was in line with maximizing sensitivity to the restricted light spectrum at greater depths and achromatic detection of overhead targets.

## Introduction

Vertebrate photoreceptors contain visual pigments that capture light to begin the process of vision^1^. Each visual pigment is composed of a protein, termed opsin, and a chromophore: retinal, the aldehyde of vitamin A_1_, or 3,4-dehydroretinal, the aldehyde of vitamin A_2_. It is the combined properties of the opsin and the chromophore that determine the absorbance of the visual pigment^2,3^. Binding of A1 retinal to an opsin will result in a visual pigment with lower wavelength of maximum absorbance (λ_max_) than conjugation to A2 3,4-dehydroretinal^3^. It is the combination of multiple cone photoreceptor types, each expressing a different predominant visual pigment, that determines the colour vision capabilities of an animal^1^. At the level of the outer retina, colour sensitivity can therefore be modulated by varying the expression of different opsins or by altering the ratio of chromophore types.

A growing number of studies suggest that many vertebrates exhibit some form of opsin modulation within individual photoreceptors. During development of human^4^, some rodents^5^ and fishes^6–9^, cone photoreceptors undergo, or are believed to undergo, opsin switches whereby the predominant opsin is replaced by another one, typically forming a visual pigment with longer λ_max_. The level of opsin co-expression that remains in the adult retina can vary substantially, as illustrated by the range found in rodents. Whereas some species co-express opsins in all cone types, forming retinal gradients of opsin expression^10,11^, others have minute numbers of co-expressing cones (<2%) and show no discernable retinal gradients^5,12^. In the mouse, opsin co-expression does not eliminate colour vision^13^ though the ventral retina, with a preponderance of S cones expressing primarily SWS1 opsin, appears to be specialized for achromatic signal detection^14^. This is because S cones are more sensitive to dark stimuli than M cones (the latter located in the dorsal retina and expressing primarily M/LWS opsin) and have greater signal gain. These characteristics suggest that the ventral retina of the mouse is specialized for detection of dark objects (such as avian predators) against the downwelling light^14^. Recordings from bipolar and ganglion cells located in the dorsal retina of the mouse show strong chromatic opponency^15,16^ indicating that this region of the retina is specialized for colour-based detection of targets on the ground.

In fishes, opsin co-expression in the adult retina appears to vary widely. In some species (e.g., salmonids^7,8^, sticklebacks^17^, milkfish^18^, winter flounder^19,20^, long tooth grouper^9^, zebrafish^21,22^), which undergo, or are believed to undergo^20^, opsin switches during development or as a function of lighting conditions, co-expression appears to be minimal or non-existent, as evaluated by microspectrophotometry and/or *in-situ* hybridization. In contrast, the adults of some cichlids show extensive, though variable, co-expression in both single and double cones^23,24^, and the predominant opsin transcript can be modulated by the rearing light environment during development^25^, the latter finding consistent with observations from black bream^26^ and guppies^27^. There is further, indirect evidence from microspectrophotometric studies that guppies^28^, anchovies^29^, and the black bream^26^ also co-express opsins in at least some cone types. Variable opsin co-expression as a function of retinal location may be a mechanism used by some fishes to increase sensitivity to different light backgrounds simultaneously^24^. This could improve the contrast of ecologically-relevant targets.

As is the case with other flatfishes, the starry flounder (*Platichthys stellatus*) hatches as a bilaterally symmetric pelagic larva^30^. Between 16 and 32 days post-hatching, at a size of ~5.9 mm, the larva starts undergoing metamorphosis, a process that involves migration of one eye to the opposite side of the head and lateral compression of the body^30^. Completion of metamorphosis, resulting in a demersal flatfish with both eyes on the same side of the body, occurs between 25 and 41 days post-hatching at a size of ~8.1 mm^30^. In the present study, we examined changes in visual pigments of the post-metamorphic starry flounder from the younger (~5 g in weight, total length ~7.5 cm) to older (~196 g in weight, total length ~18–23 cm) juvenile. During this period, spanning more than two years of growth, the fish in nature progresses from life in shallow bays (1–20 m in depth) to deeper oceanic waters along the coastal shelf (>20 m in depth)^31^. This is accompanied by a change in the light environment from full spectrum (~310–850 nm) downwelling light of high intensity (~10^19^–10^21^ photons m^−2^s^−1^) near the surface, that is also direction-dependent, to a narrower wavelength (~450–600 nm), lower intensity (<10^17^ photons m^−2^s^−1^ beyond 20 m depth) downwelling light with minimal sidewelling or upwelling light^32^. The post-metamorphic starry flounder settles on the ocean floor and progresses from consumption of translucent planktonic copepods to mysids^31^, amphipods, and other invertebrates, some of which can be located overhead. In turn, it is preyed upon by birds, marine mammals and bigger fish that scan the ocean floor from above^33^. The visual ecology of the starry flounder (and of flatfishes in general) is therefore very different from that of the few pelagic fishes that have been examined for opsin co-expression.

In a previous investigation, eight visual pigments were measured, using microspectrophotometry, that corresponded to seven cone opsins and one rod opsin (whose transcripts were quantified by digital PCR) in the retina of the young, post-metamorphic starry flounder (~5–15 g in weight)^34^. The correspondence between visual pigments and opsin types was as follows: UV(369)-SWS1, S(415)-SWS2B, S(437)-SWS2A2, S(456)-SWS2A1, M(527)-RH2A1, M(545)-RH2A2, L(557)-LWS, and rod (507)-RH1. That study, however, did not document incidences of co-expression or modulation of opsin expression as the fish grew. Co-expression of opsins has not been reported in the retinas of other flatfishes, such as the winter flounder^19,20^ or the Atlantic halibut^35^, which appear to have only five opsins. Thus, whether some form of opsin modulation within individual photoreceptors occurs in flatfish retinas is unknown. Here, we investigated this question using microspectrophotometry and high performance liquid chromatography with an emphasis on understanding visual pigment adaptations for a demersal life style. In turn, our findings help to evaluate variable opsin co-expression as a general mechanism for light sensitivity-tuning in vertebrate retinas.

## Results

### Visual pigments and chromophores in the retina of large juvenile starry flounder

**Figure 1:**
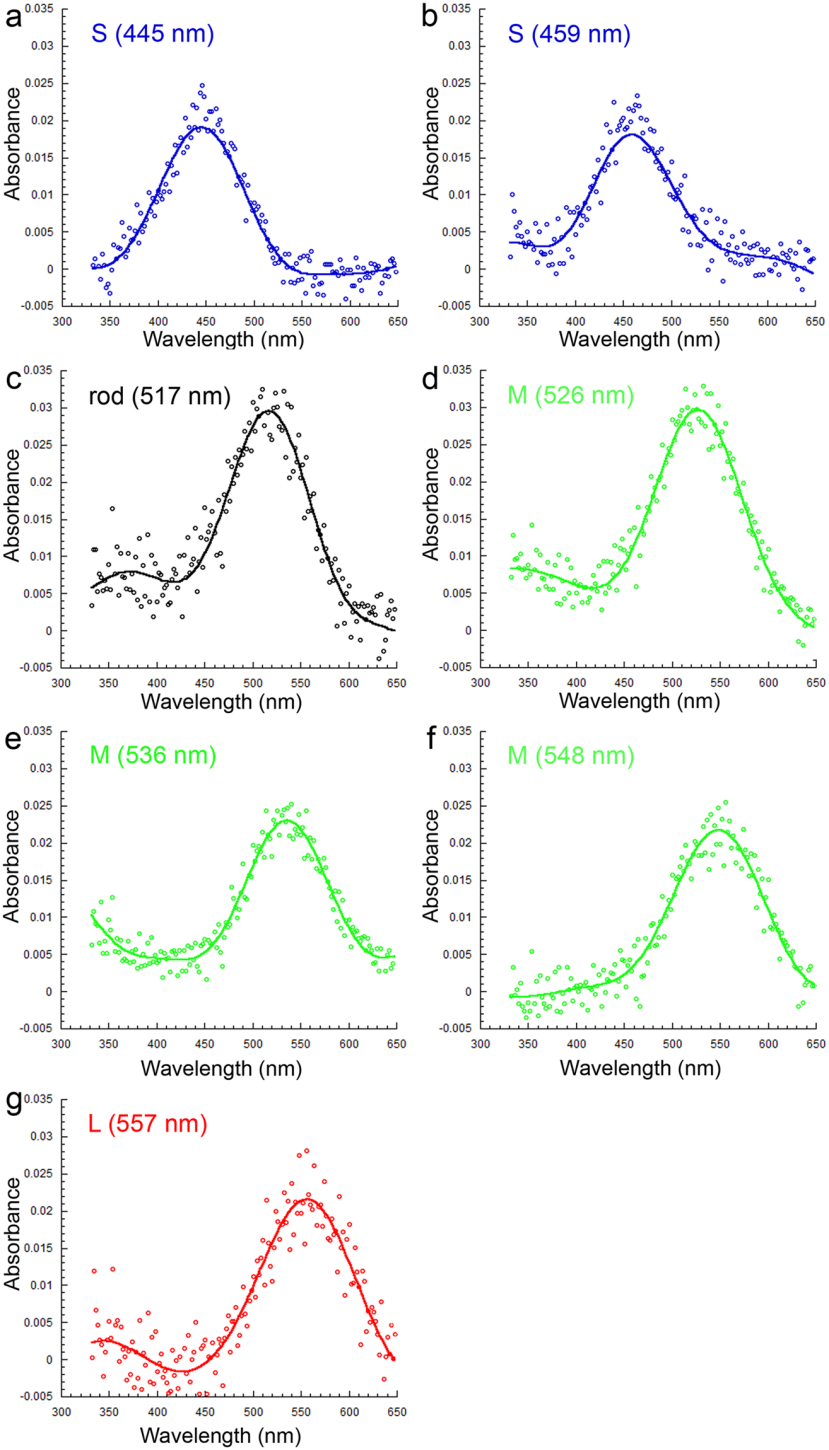
Visual pigments of large juvenile starry flounder (mean weight of 196.2 g). Mean absorbance spectra from a total of 5–9 cells from 3 fish showing: (**a**,**b**) short wavelength (S) visual pigments, (**c**–**f**) middle wavelength (M) visual pigments [the rod visual pigment is depicted in (**c**)], and (**g**) the long wavelength (L) visual pigment. The λ_max_ of each pigment is indicated on the corresponding graph between parentheses.

Using microspectrophotometry, we found six cone visual pigments and one rod visual pigment in the large post-metamorphic juvenile starry flounder (mean weight: 196.2 g). These consisted of two S visual pigments with λ_max_ at 445 nm (n = 19) and 459 nm (n = 17), three M visual pigments with λ_max_ at 526 nm (n = 14), 536 nm (n = 5) and 548 nm (n = 18), one L visual pigment with λ_max_ at 557 nm (n = 5), and a rod visual pigment with λ_max_ at 517 nm (n = 16; means from n photoreceptors from 3 fish; Fig. 1). All but the S (445), M (536) and the rod visual pigment were also present in younger fish (Table 1).

**Table 1:** Statistics of visual pigments in the mean λ_max_ range 437–557 nm for large and small post-metamorphic starry flounder. In addition to the L (557), the following pigments had statistically similar λ_max_: S (459) and S (456), M (526) and M (527), and M (548) and M (545). HBW, the bandwidth at half maximum absorbance, was also predicted for the visual pigments in large starry flounder using a semi-empirical model based on opsin conjugation to A1 retinal chromophore^3^. Experimental results are the mean ± SD of n photoreceptors from three fish. The rod results are indicated in the last column.

Large starry flounder (mean weight: 196.2 g)							
λ_max_ ± S.D. (nm)	445 ± 8.4	459 ± 3.3	526 ± 4.1	536 ± 6.8	548 ± 5.0	557 ± 5.7	517 ± 3.5
HBW ± S.D. (cm^−1^)	5091 ± 461	4671 ± 507	3933 ± 313	4125 ± 384	3673 ± 298	3493 ± 263	4046 ± 258
n	19	17	14	5	18	5	16
HBW-predicted (cm^−1^)	4838	4763	4100	4015	3906	3774	4206
Small starry flounder (mean weight: 5.4 g)							
λ_max_ ± S.D. (nm)	437 ± 8.3	456 ± 6.2	527 ± 4.9		545 ± 6.5	557 ± 10	507 ± 3.9
HBW ± S.D. (cm^−1^)	4889 ± 439	4818 ± 266	4142 ± 308		4014 ± 334	3733 ± 293	4072 ± 205
n	18	23	16		13	6	21

**Figure 2:**
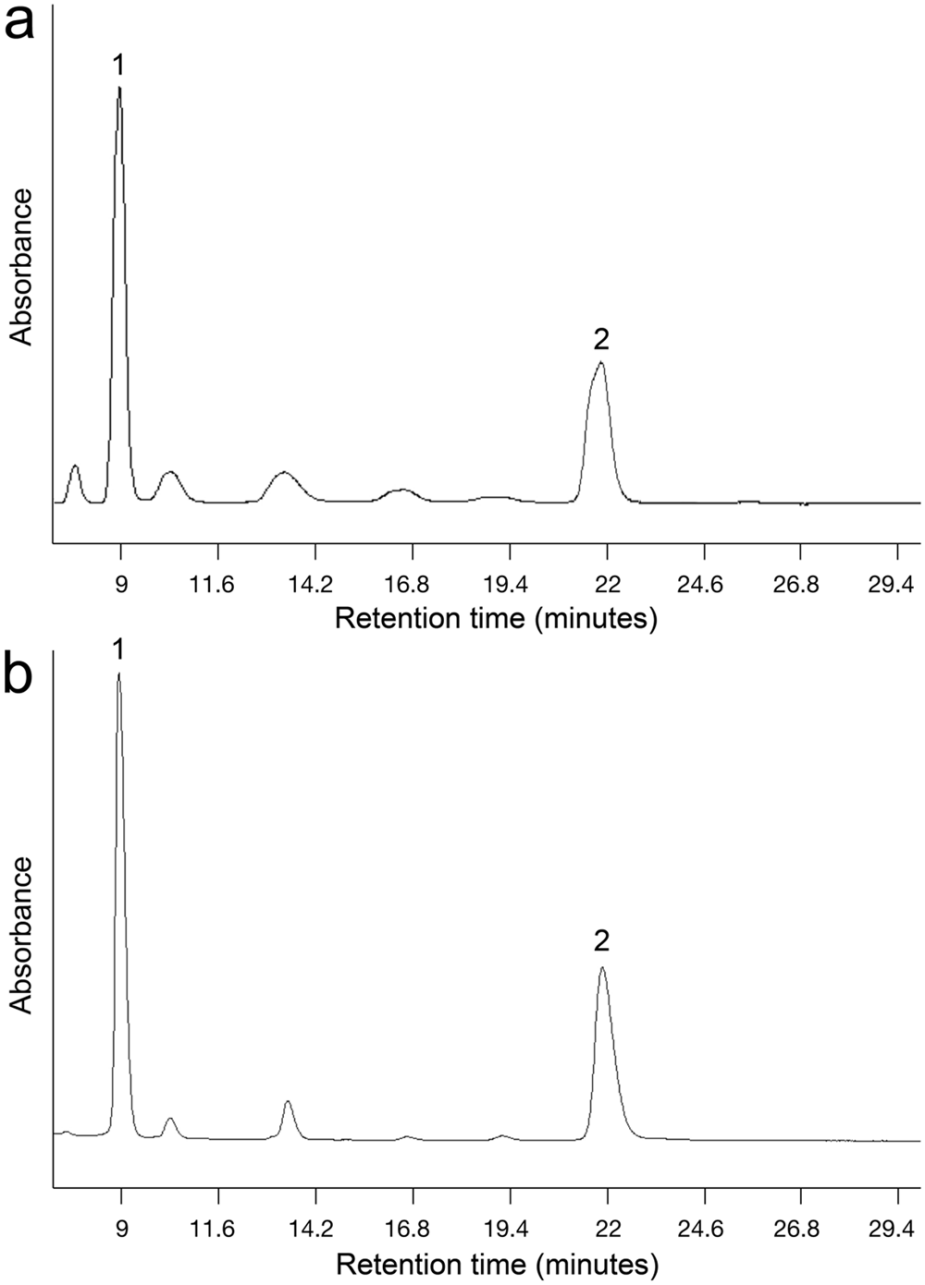
Chromophores in the retina of large juvenile starry flounder (mean weight of 121 g). Representative high performance liquid chromatography absorbance profiles of: (**a**) large starry flounder retina extract, and (**b**) the all-trans retinal (vitamin A1) standard. Peak 1 corresponds to all-trans retinal and peak 2 to anti-all-trans retinal.

**Figure 3:**
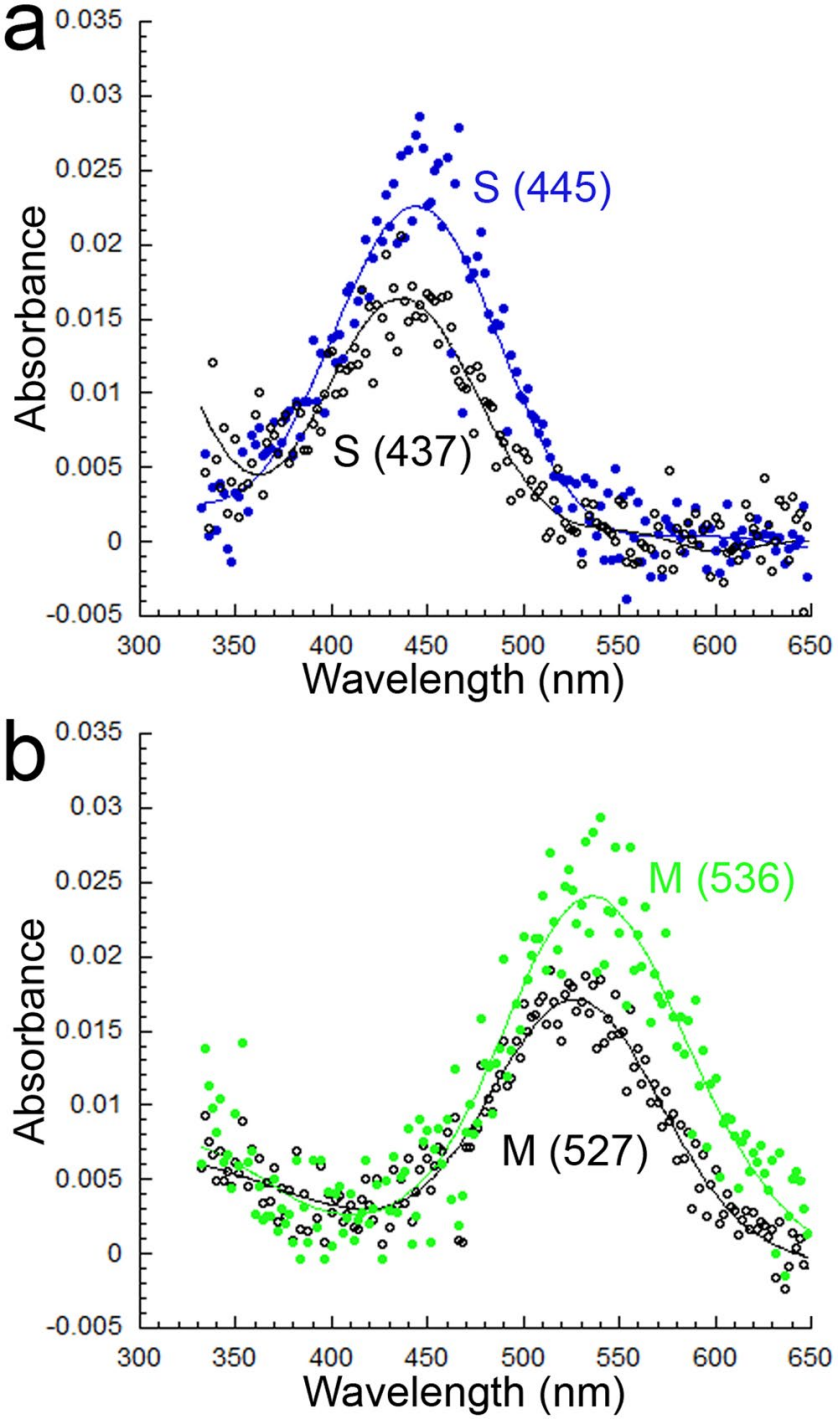
Visual pigments and their bleached photo-products. (**a**) Bleaching of the S (445) visual pigment gave rise to an S (437) visual pigment. (**b**) Bleaching of the M (536) visual pigment gave rise to an M (527) visual pigment. Each trace is the average of 4 records.

Both the S (445) and M (536) visual pigments had bandwidths at half maximum (HBW) that were greater than those associated with the rest of the S or M visual pigments, respectively, regardless of fish size (Table 1). These bandwidths were also greater than those predicted for such pigments using empirical models based on opsin conjugation to A1 retinal chromophore^3^ (Table 1). Such results suggested the contribution of multiple opsins to the S (445) and M (536) visual pigment absorbance curves since analyses of retinal extracts using High Performance Liquid Chromatography revealed the same A1 retinal chromophore as present in younger fish^34^ (Fig. 2). Bleaching of the S (445) visual pigment with 480 nm light for 2 minutes resulted in a new absorbance curve peaking at 437 nm (Fig. 3a). This result indicates that the S (445) visual pigment consisted of two visual pigments: the S (437) and the S (456), the latter bleached by the 480 nm light. Likewise, bleaching of the M (536) visual pigment with 580 nm light for 2 minutes resulted in a new absorbance curve peaking at 527 nm (Fig. 3b) indicating that the M (536) visual pigment was a combination of the M (527) and M (545) visual pigments, the latter bleached by the 580 nm light.

The HBW of the rod visual pigment was commensurate with that expected from absorbance by a single visual pigment bound to A1 retinal^3^. This is because vitamin A1-based visual pigments with peak absorbance around 500 nm have HBWs in the range 4000–4200 cm^−1^ whereas those binding A2 3,4-dehydroretinal have HBWs in the range 4800–4900 cm^−1^ ^2^. The λ_max_, however, suggested that the rod opsin was conjugated to a combination of vitamin A1 and A2 chromophores^3^. Since only vitamin A1 chromophore was present in the retina, the rod photoreceptor in the larger juvenile had to be different from that in the smaller fish. Sequencing of approximately 80% of the rod opsin in the larger juvenile and comparisons to a published sequence for adult starry flounder from the Sea of Japan showed no difference with that previously reported for the 5–15 g fish^34^ except at positions 217 (A) and 868 (V). These substitutions are not located at sites associated with large shifts in spectral tuning of rod photoreceptors^36^. Thus, at present, the ~10 nm long wavelength shift of the rod visual pigment in the larger juvenile cannot be explained with available molecular data. It is possible that a different rod opsin (i.e., a gene not present in the transcriptome of the small juvenile^34^) is co-expressed in rod photoreceptors of the larger juvenile; additional RH1 opsins have recently been reported in other fishes^37,38^.

### Visual pigment gradients within single and double cones of the small juvenile

**Figure 4:**
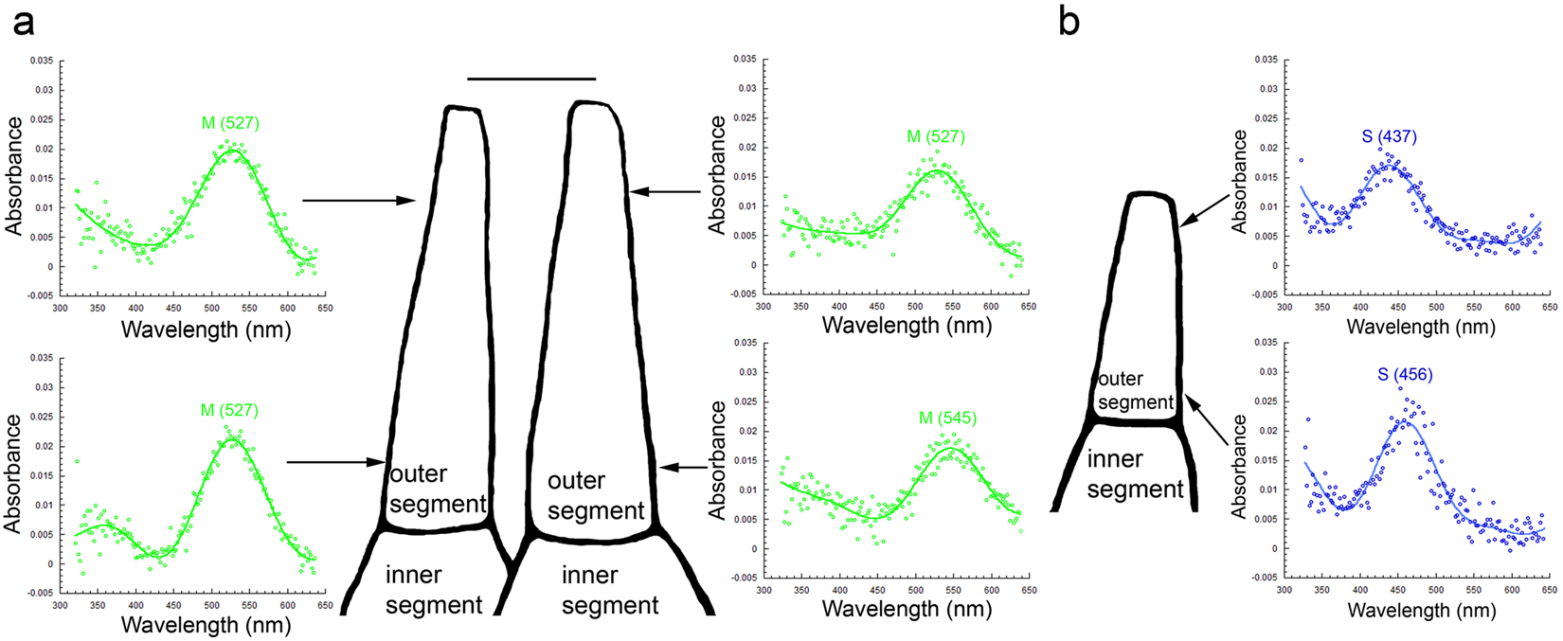
Co-expression of two distinctly measurable visual pigments in the cones of small starry flounder (mean weight of 5.4 g). (**a**) Some double cones had an M (527) visual pigment in one cone member and two visual pigments in the other member: an M (545) at the base and an M (527) at the tip of the outer segment. (**b**) Some single cones had an S (456) visual pigment at the base and an S (437) at the tip of the outer segment. Each trace is the average of 5 records. The magnification bar in (**a**) corresponds to 5 µm [and holds for (**a**) and (**b**)].

**Figure 5:**
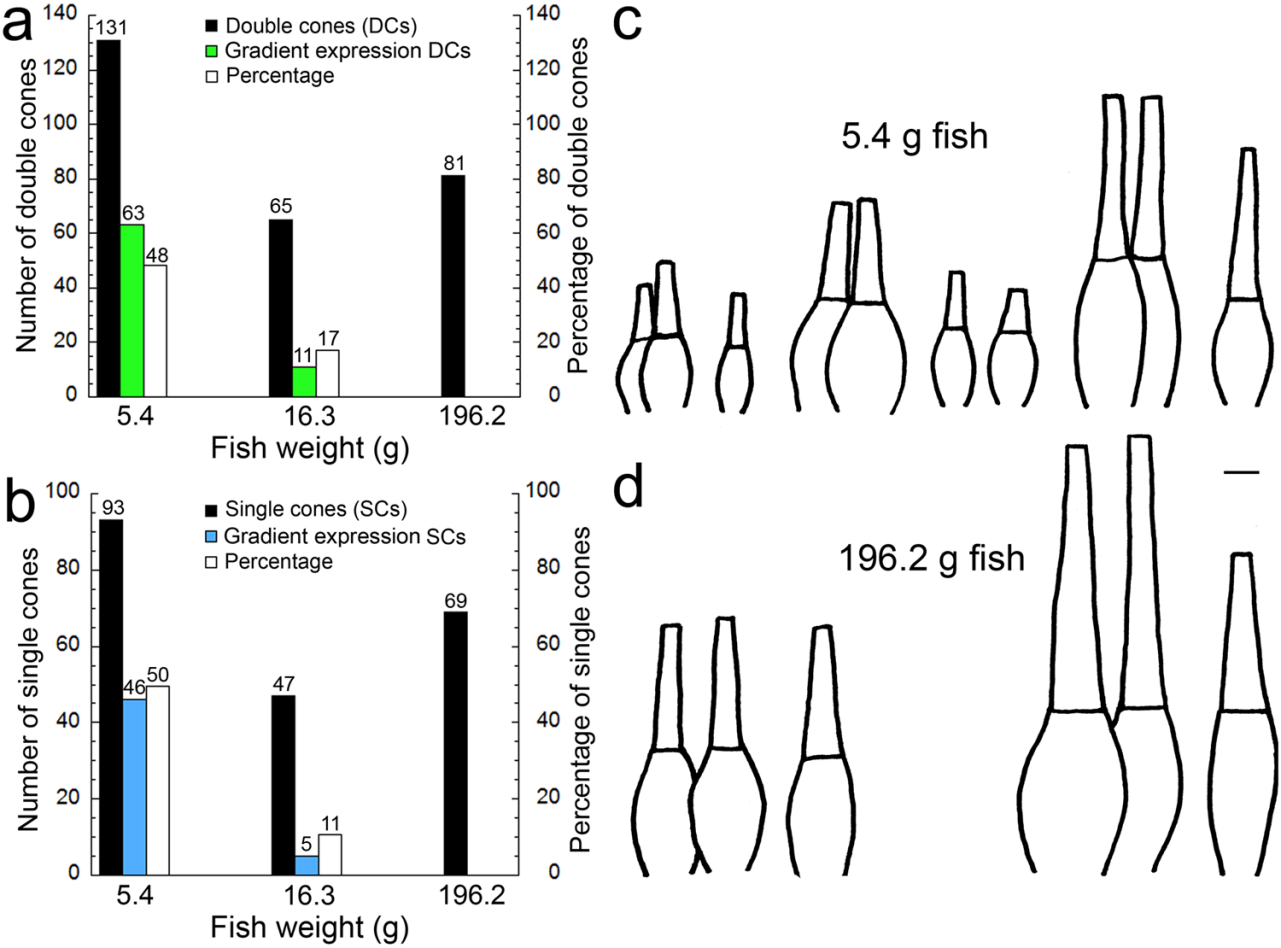
Proportions of double and single cones that express a visual pigment gradient and microspectrophotometric records of morphological cone types observed. (**a**) Bar graph showing the number of double cones measured by microspectrophotometry at the various fish sizes analyzed, the number of these cones that showed a gradient of visual pigment content in one member, and their percentage. Numbers on bar graphs correspond to their y axis values and are in the appropriate units: numbers or percentage (see colour legend on Figure). (**b**) Similar graph to (**a**) but for single cones. (**c**,**d**) Traces of representative cones, without synaptic apparatus, obtained from microspectrophotometric records of small starry flounder (**c**) and large starry flounder (**d**). In each case, the largest cones were observed in the ventral retina and the smallest cones in the dorsal retina; intermediate-size cones were observed in the smaller starry flounder and were associated with the central retina. The magnification bar in (**c**) corresponds to 5 µm [and holds for (**c**) and (**d**)].

Microspectrophotometric observations revealed within photoreceptor gradients of visual pigment occurrence in the population of double and single cones in the smaller fish (mean weight: 5.4 g). These gradients were characterized by two distinctly-measurable single visual pigments, at the base and tip of the outer segment respectively. Among the double cones, some had a single M (527) visual pigment in the outer segment of one member, whereas the other member housed an M (545) visual pigment at the base and an M (527) at the tip (Fig. 4a). Among the single S cones, some had an S (456) visual pigment at the base and an S (437) at the tip of the outer segment (Fig. 4b). The proportion of cones exhibiting a visual pigment gradient along the outer segment decreased from ~50% in the smaller juvenile to zero in the large juvenile, with a ~10–15% proportion present in intermediate size fish (mean weight: 16.3 g) (Fig. 5a,b). These spatially segregated visual pigments along an outer segment were primarily associated with larger cones, which have been localized to the ventral retina both by histology and microspectrophotometry^34^ (Fig. 5c,d). From our microspectrophotometry observations, the scaling of cone types with growth between ventral and dorsal retina appeared similar, though the variety of sizes was smaller in the larger juvenile (Fig. 5c,d).

### Light with depth and visual sensitivity thresholds

**Figure 6:**
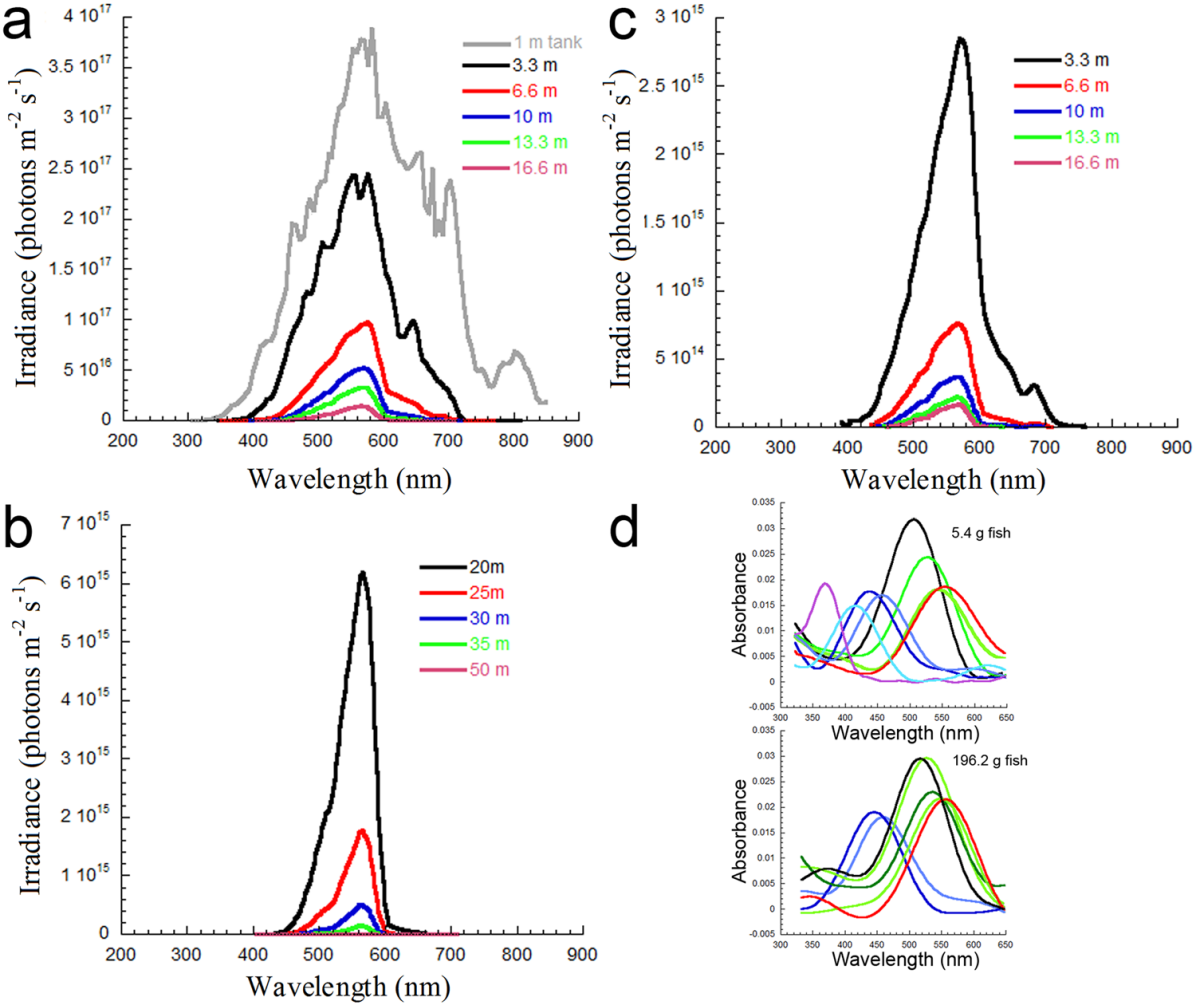
Spectral irradiance in temperate coastal waters inhabited by starry flounder, and visual pigments in the small and large juvenile. (**a**) Downwelling spectral irradiance in the holding tank at 1 m depth, and in the ocean for depths between 3.3 and 16.6 m, measured with an underwater spectroradiometer. (**b**) Computed spectral irradiance for depths between 20 and 50 m using extinction coefficients derived from ocean data in (**a**). (**c**) Upwelling spectral irradiance measured for depths between 3.3 and 16.6 m. (**d**) Fourier derived curve fits to the visual pigment data obtained for small and large starry flounder.

The downwelling light spectrum at 1 m depth in the tank where the fish were held was broadband (310–850 nm, Fig. 6a) and resembled the spectra previously reported for coastal temperate surface waters^32^. In nature, the light spectrum peaked in the range 550–560 nm at depths below 10 m and between 565–570 nm at greater depths (Fig. 6a,b). The downwelling spectrum at 3.3 m depth was 335–810 nm and reduced to 405–710 nm by 16.6 m depth. The upwelling spectrum was 390–760 nm at 3.3 m depth and 460–610 nm at 16.6 m depth (Fig. 6c). The downwelling irradiance at each depth was, on average, 116 times that of the upwelling irradiance. Based on a threshold irradiance of 10^13^ photons m^−2^ s^−1^, required to elicit physiological and behavioural visual responses in several fishes and crustaceans^39,40^, the predicted limiting depth for visual perception by starry flounder in this environment was 51 m. The analogous depth from upwelling light was 36 m.

Of the seven cone visual pigments found in the small starry flounder^34^, only the UV (369) and the S (415) were not detected by microspectrophotometry in the larger juvenile. The absorbance spectrum of the UV (369) could reach up to 450 nm whereas that of the S (415) could extend up to 500 nm (Fig. 6d). Based on downwelling integrated irradiances in the range 300–450 nm and 300–500 nm, the UV (369) visual pigment could only be stimulated by light, and thus contribute to vision, to a depth of 25 m, and the S (415) to a depth of 32 m.

## Discussion

The number of cones that exhibited a gradient of visual pigments along the outer segment decreased from ~ 50% in the smallest starry flounder to 0% in the largest starry flounder. Such a gradient was characterized by the presence of two visual pigments, one dominating the base of the outer segment with longer λ_max_, and the other present at the tip of the outer segment with shorter λ_max_. These observations, which were common to central and more peripheral areas of the retina (the former present in the small juvenile and the latter grown afterwards), suggest that a proportion of the double cone population switched phenotype from equal M (527)/M (527) to unequal M (527)/M (545) as the fish grew. Likewise, a segment of the single cone population changed from S (437) to S (456). Such replacement of visual pigments suggests switches in opsin expression as vitamin A1 was the only chromophore found in the retina. Switches in opsin expression have also been reported in the single cones of some rodents^5^, juvenile salmonid fishes^7,8^, and zebrafish^21,22^, and are suspected to take place during development of winter flounder^20^, the black bream^6^, the long tooth grouper^9^, and human^4^. In all these species, the remaining visual pigment has longer λ_max_ than the one replaced. The fish in our experiments were kept in captivity and experienced a light spectrum that was broader and more intense than what they would have experienced in nature (Fig. 6a). Because environmentally-induced changes in the types of opsins expressed are commonly associated with alterations in the light spectrum experienced by the fish^17,25–27^, our results suggest that the modulation observed was inherited. As such, the opsin changes are likely representative of what occurs in nature.

Our absorbance measurements failed to detect gradients of opsin expression within cone outer segments in large starry flounder. Nonetheless, some single cones and one member of some double cones expressed a mixture of two visual pigments. This resulted in cones that had intermediate peak absorbance at 445 nm (for the single cones) and 536 nm (for one member of the double cones). Co-expression of opsins in single and double cones has been reported in some species of cichlids, with large variation between retinal sectors and individuals^24^. The advantages of such co-expression are unclear. One hypothesis is that variable opsin expression with retinal sector maximizes simultaneous detection of targets against different light backgrounds^23^.

Cichlids are pelagic fishes which experience heterogeneous light backgrounds with line of sight. The light environment surrounding a bottom-dwelling fish like the starry flounder is much more homogeneous, especially with increasing depth. Our calculations show that in local temperate coastal waters, a total irradiance of 10^13^ photons m^−2^ s^−1^ is no longer present deeper than 51 m. Furthermore, this threshold irradiance, which is necessary to elicit optic nerve or behavioural responses in other fishes^40^ and crustaceans^39^, is only present as part of the downwelling light field at depths beyond ~36 m. Thus, large starry flounder residing deeper than 36 m would only receive downwelling illumination, and of restricted spectrum (~480–600 nm). Starry flounder are generally caught at depths up to 50 m^31,41^, which corresponds almost exactly to the threshold depth for visual function derived from our calculations, suggesting that vision is the limiting factor in the depth distribution of this species.

Seven cone visual pigments were found in the smaller juvenile starry flounder and only five in the larger juvenile, since one of each of the S and M pigments were a mixture of two others. Such reduction in visual pigments is in line with life at greater depths, where light becomes restricted to the downwelling direction and the spectrum centred around 560–570 nm. Our theoretical calculations show that, beyond a depth of ~32 m, the complement of visual pigments in the small starry flounder would not provide any extra sensitivity compared to that obtained from the restricted complement found in the larger starry flounder. Thus, the failure to find UV (369) and S (415) visual pigments in the large starry flounder agrees with what may be expected from progression to greater depths. The overall increase in M (545) and S (456) visual pigments in the large juvenile is also consistent with maximizing sensitivity to a restricted light field with depth. Upregulation of the corresponding longer wavelength RH2 and SWS2 opsin transcripts and decreases in SWS1 and SWS2B occur with growth of another flatfish, the barfin flounder^42^. Other fish species whose adults frequent depths beyond 30 m, like the Atlantic cod^43^, also have a preponderance of S and M visual pigments in the retina.

Like cichlids, the large starry flounder co-expresses visual pigments although it most often resides in an environment that is chromatically homogeneous. Thus, modulation of opsin content for simultaneous maximal chromatic contrast of targets against different light backgrounds is unlikely to be a major ecological function associated with the adult starry flounder retina. With the exception of brief incursions into shallow bays for the purpose of reproduction during the spring^41^, where colour vision may play some ecological role, we suspect that the visual system of the adult starry flounder is optimized for the detection of silhouettes against the downwelling light. As such, it is tempting to draw analogies with the ventral retina of the mouse, which also needs to detect achromatic targets (such as avian predators) above the animal. In the mouse, co-expressing S cones in the ventral retina exhibit preference for dark over light stimuli, and this cannot be explained by visual pigment properties^14^. It is hypothesized that some other component of the phototransduction cascade or synaptic apparatus, or negative feedback from horizontal cells, shapes the physiological output of these ventral cones, which is different from that of cones in the rest of the retina^14^. If this finding can be replicated in other species, the possibility for colour vision or its modulation may not be a simple function of expression of multiple opsins/visual pigments within or between cone types, but of additional molecular components that determine whether cone photoreceptors contribute to chromatic or achromatic pathways. It is therefore possible that at least the ventral retina of the adult starry flounder is also wired for achromatic vision, and that co-expression of opsins in multiple S and M spectral cone types is best suited for this functionality. In this regard, the OFF response (achromatic pathway) of some other fishes, like the salmonid fishes^40^ and goldfish^44^, consists of variable input from S, M, and L cones, with the predominant input matching the most transmitted region of the light spectrum. The preponderance of S an M visual pigments in the large starry flounder, with absorbances that overlap the most transmitted region of the spectrum, suggest that this species may share a similar OFF response to that of salmonid fishes.

## Materials and Methods

### Animals

Young post-metamorphic starry flounder were collected by seine net from Willows Beach (Victoria, British Columbia, Canada) and reared for >2 years at the University of Victoria Aquatic Facility. The animals were housed in a 1.5 m deep outdoor tank and experienced the natural daylight cycle (Fig. 6a). Temperature of the re-circulating water in the facility mirrored (though in dampened form) that outside and ranged from 10 °C in the winter to 15 °C in the summer. Fish were sacrificed between 13:00–14:00 hrs, during the daylight portion of their circadian rhythm. All animal use was approved by the Animal Care committees of Simon Fraser University (protocol # 1126B-10) and the University of Victoria (protocol # 2013–005), which abide by regulations set by the Canadian Council for Animal Care.

### Animal experimentation approval

All animal use was approved by the Animal Care committees of Simon Fraser University (protocol # 1126B-10) and the University of Victoria (protocol # 2013–005), which abide by regulations set by the Canadian Council for Animal Care.

### Microspectrophotometry

Measurements of visual pigment absorbance were obtained from isolated photoreceptors using a computer-driven dichroic microspectrophotometer (DMSP). The instrument, measuring procedure and analysis of absorbance curves has been detailed in previous publications^45,46^. For this work, we cut each retina of the large starry flounder into four quarters intersecting at the position of the embryonic fissure head, which in these animals defines the centre of the retina. Each of the quarters was then cut obliquely (i.e., in rough alignment with the retinal perimeter) to create a small retinal triangle of ~2 mm in height, and another, parallel cut ~4 mm from the first that comprised the remainder of the quarter but without the periphery (retinal diameter was ~15 mm in the large starry flounder). Small triangles from the centre of the retina were analyzed separately from the remainder of the retina. This procedure was carried out to differentiate trends in visual pigments in the central area of the retina (which was present in the small juveniles, since their retinal diameter was 3.5–4 mm) from areas closer to the periphery (which would comprise new retina grown following the small juvenile stage). Three size groups of fishes were analyzed by microspectrophotometry with the following characteristics (weight ± SD and total length ± SD): small juvenile (5.4 g ± 0.3 g, 7.8 cm ± 0.2 cm, n = 5), intermediate juvenile (16.3 g ± 2.6 g, 11.9 ± 0.4 cm, n = 4), and large juvenile (196.2 g ± 7.5 g, 22.6 cm ± 1.6 cm, n = 3).

### High performance liquid chromatography (HPLC)

Following dark-adaptation for 3 hours, two large starry flounder (mean weight: 121 g) were killed and the retinas removed from the eyecups. Each retina was then placed in a 1.5 ml eppendorf tube and frozen in liquid nitrogen until processing for presence of chromophore types: A1 retinal (vitamin A1) and A2 3,4-dehydroretinal (vitamin A2). Retinas were homogenized by sonication in a mixture containing 60 µl of 1.92 M hydroxylamine sulfate (neutralized with 1 N KOH) and 300 µl of methanol. The homogenate was cooled on ice, following which 300 µl of dichloromethane and 150 µl of ddH_2_O were added and the mixture shaken vigorously. After the addition of 600 µl of n-hexane, the solution was centrifuged at 1000 g for 5 minutes at 4 °C. The dichloromethane/hexane (top) layer was removed and placed in a 1.5 ml eppendorf tube. This procedure was then repeated on the remaining, lower layer, after which the second extract was combined with the first and evaporated under vacuum for 1 hour. The resulting, dried extract was dissolved in 100 µl of n-hexane and injected into a 5 µm YMC-Pack Silica 2.1 × 250 mm column (YMC America) attached to an HPLC system. The mobile phase running through the column flowed at a rate of 0.8 ml per minute and contained 7% ether and 0.075% ethanol in n-hexane. The computer-driven detector monitored absorbance at 360 nm and 400 nm and produced chromatograms which permitted identification of vitamin A1 and A2 oximes based on retention time in comparison with results from an all-trans-retinal (A1) standard (Sigma). There is no commercially available dehydroretinal (vitamin A2) standard but the technique will readily detect vitamin A2 oxime if present in the sample, as has been demonstrated previously^34^.

### Light measurements and spectral irradiance integrations

Irradiance light measurements were acquired with a Licor LI-1800 underwater spectroradiometer (Lincoln, Nebraska), either by hand immersion of the sensor in the holding tank or using SCUBA in the ocean. The spectroradiometer was positioned with the cosine collector sensor pointing upwards (downwelling light) or downwards (upwelling light) at multiple depths from 3.3 m to 16.6 m in Saanich inlet (Vancouver Island, British Columbia, Canada) with the ocean floor located at 150 m. Based on extinction coefficients for the spectrum 300–850 nm, calculated from downwelling measurements at 13.3 and 16.6 m depth, we derived spectral irradiance functions up to 100 m depth. Integrated irradiances were used to compare downwelling and upwelling light levels, and to estimate threshold depths for starry flounder vision. The latter were based on the fact that ~10^13^ photons m^−2^ s^−1^ is the irradiance required to elicit organ or whole animal visual responses in a variety of fishes and crustaceans^39,40^ that also inhabit these waters.

### Availability of data

Data are available in the manuscript and as requested from the corresponding author.
